# Effect of hawthorn standardized extract on flow mediated dilation in prehypertensive and mildly hypertensive adults: a randomized, controlled cross-over trial

**DOI:** 10.1186/1472-6882-12-26

**Published:** 2012-03-29

**Authors:** Gary N Asher, Anthony J Viera, Mark A Weaver, Rosalie Dominik, Melissa Caughey, Alan L Hinderliter

**Affiliations:** 1 Department of Family Medicine, University of North Carolina, Chapel Hill, NC, USA; 2 Department of Medicine, University of North Carolina, Chapel Hill, NC, USA; 3 Department of Biostatistics, University of North Carolina, Chapel Hill, NC, USA; 4 Division of Cardiology, University of North Carolina, Chapel Hill, NC, USA

**Keywords:** Hawthorn, Crataegus, hypertension, prehypertension, flow mediated dilation, Phase I

## Abstract

**Background:**

Hawthorn extract has been used for cardiovascular diseases for centuries. Recent trials have demonstrated its efficacy for the treatment of heart failure, and the results of several small trials suggest it may lower blood pressure. However, there is little published evidence to guide its dosing. The blood pressure lowering effect of hawthorn has been linked to nitric oxide-mediated vasodilation. The aim of this study was to investigate the relationship between hawthorn extract dose and brachial artery flow mediated dilation (FMD), an indirect measure of nitric oxide release.

**Methods:**

We used a four-period cross-over design to evaluate brachial artery FMD in response to placebo or hawthorn extract (standardized to 50 mg oligomeric procyanidin per 250 mg extract). Randomly sequenced doses of hawthorn extract (1000 mg, 1500 mg, and 2500 mg) and placebo were assigned to each participant. Doses were taken twice daily for 3 1/2 days followed by FMD and a 4-day washout before proceeding to the next dosing period.

**Results:**

Twenty-one prehypertensive or mildly hypertensive adults completed the study. There was no evidence of a dose-response effect for our main outcome (FMD percent) or any of our secondary outcomes (absolute change in brachial artery diameter and blood pressure). Most participants indicated that if given evidence that hawthorn could lower their blood pressure, they would be likely to use it either in conjunction with or instead of lifestyle modification or anti-hypertensive medications.

**Conclusion:**

We found no evidence of a dose-response effect of hawthorn extract on FMD. If hawthorn has a blood pressure lowering effect, it is likely to be mediated via an NO-independent mechanism.

**Trial Registration:**

This trial has been registered with ClinicalTrials.gov, a service of the U.S. National Institutes of Health: NCT01331486.

## Background

Hawthorn (*Crataegus *spp.) is a thorny shrub that grows commonly in northern temperate regions around the world. Typically the leaf and flower, berry, or a combination of all three are consumed as a powder, tea, or liquid extract. Its therapeutic use is reported as early as the 1^st ^century BCE, and references to its use in cardiovascular diseases date to the 1600's [1].

The chemical constituents considered to be the primary bioactive components of hawthorn are the flavonoids and oligomeric procyanidins (OPCs) [2]. Hawthorn extracts have been shown to enhance release of nitric oxide (NO) from vascular endothelium causing vasodilation, which appears to be associated with the OPC-rich fraction of hawthorn extract [3-5].

While traditional indications for use of hawthorn include asthma, diabetes, and neurasthenia [6], the most substantial evidence for its benefit resides in treatment of mild to moderate heart failure (HF) [7,8]. As an adjunct to conventional treatment in patients with HF (New York Heart Association classes I - III), hawthorn extract may provide additional benefit in symptom control (eg. fatigue, shortness of breath) and physiologic outcomes (eg. maximal work load tolerance, exercise tolerance, pressure-heart rate product) [8]. Several small clinical trials with hawthorn have demonstrated modest blood pressure reduction [9-11], and a few preclinical studies have shown reductions in total cholesterol, low-density lipoprotein, and ApoB synthesis [12-16]. However, there are no published evaluations of human dose-response to hawthorn to guide its dosing. We sought to evaluate the dose-response effect of a hawthorn extract standardized to OPC concentration on flow-mediated dilation of the brachial artery (FMD), an indirect measure of NO release [17].

## Methods

### Participants

English-speaking adults aged 18 years and older with recent average ambulatory systolic blood pressure (BP) between 120-155 mmHg and diastolic BP between 80-95 mmHg were eligible for enrollment. People using any antihypertensive medications or tobacco products, as well as those with a prior diagnosis of diabetes mellitus, coronary artery disease, severe aortic stenosis, idiopathic hypertrophic subaortic stenosis, or upper extremity vascular obstruction were excluded. Pregnant or breast-feeding women, and women using estrogen-containing birth control methods, were also excluded. All participants were instructed to forego the use of dietary supplements such as vitamins C and E, fish oil, niacin, arginine, and over-the-counter decongestants and non-steroidal anti-inflammatory agents for the duration of the study. Participants were asked to refrain from alcohol, vigorous exercise, and use of phosphodiesterase inhibitors prior to study visits. Participants experiencing any respiratory or viral illness associated with fever, as well as any acute inflammatory conditions, were suspended from involvement in the study until the acute illness had resolved. The Institutional Review Board at the University of North Carolina approved the research protocol, and all study participants gave written consent prior to participation in the study.

### Study design

We used a randomized, placebo-controlled, double-blind, four-period crossover design. Each participant had brachial artery FMD measured at baseline and after each dosing period. Capsules containing 250 mg of hawthorn standardized extract (HSE) and a sufficient number of matching placebo capsules were combined to create four dosage levels: placebo, 1000 mg, 1500 mg, and 2500 mg. We chose two doses (ie. 1000 mg [200 mg OPC], 1500 mg [300 mg OPC]) to approximate the previously reported doses from heart failure trials (ie. 900 mg [168 mg OPC], 1800 mg [337 mg OPC]) [18-20] and a third dose (2500 mg [500 mg OPC]) above those previously tested.

Capsules were prepackaged into single-dose cups each containing five capsules that were taken twice daily (e.g., at 1500 mg, each dose cup contained 3 HSE and 2 placebo capsules). Within each period, doses were taken for three consecutive days followed by a final dose on the morning of the FMD measurement for a total of seven consecutive doses. There was a minimum 3 1/2 day washout period between dosage levels. The washout period was timed to be greater than 5 half-lives of the hawthorn anthocyanidin epicatechin (t_1/2 _= 80 min) [21]. Four random dose sequences were created using a Williams design [22] and participants were randomly assigned to sequence following a schedule generated by an independent statistician; allocation concealment was accomplished using consecutively numbered sealed opaque envelopes. The study pharmacist opened envelopes in sequential order and dispensed the appropriate dose cups; all other study personnel were blinded until all participants completed follow-up.

HSE (Hawthorn Supreme Liquid Phyto-Caps) and matching placebo were obtained from Gaia Herbs, Inc., Brevard, NC. The extract was produced from a single, certified organic study lot grown at the Gaia Herb Farm and was processed using a standard protocol. Extraction solvents included grain alcohol and water. Approximately 1000 mg of whole leaf and flower was used to produce a single 250 mg capsule standardized to 50 mg oligomeric procyanidins, which was verified using ultraviolet-visible spectroscopy. Placebo caps were matched for color and liquid viscosity.

### Procedures

FMD was measured using a 12.5 MHz imaging probe interfaced with an ATL HDI 5000 ultrasound machine. A single experienced technician performed all FMD measurements under standard conditions (i.e., participants fasted overnight, measurements were taken in the morning) [23].

Participants were asked to lie supine on a stretcher in a quiet private room, and a pneumatic tourniquet was placed around the right lower arm distal to the brachial artery. Gated baseline images of the brachial artery and Doppler flow profiles were acquired after 15 minutes of supine rest. The pneumatic cuff was then inflated to a pressure of 200 mm Hg for 5 minutes and increased flow (i.e., hyperemia) induced by sudden cuff deflation. A second Doppler scan was performed immediately following deflation, with imaging of the brachial artery for 90 seconds. Digital images of the artery were stored for subsequent off-line quantification.

Doppler measurements of brachial arterial flow during imaging at baseline and during hyperemia were quantified by the HDI 500 ATL ultrasound machine. The Hi-Q trace tracked the flow curves and quantified the velocity-time integral (VTI) and peak systolic velocity under each condition.

### Outcome measures

Brachial arterial diameter (BAD) was measured from the lumen-intimal interfaces of the proximal and distal arterial walls using customized software (Brachial Tools, Medical Imaging Applications, LLC). Data from at least three consecutive end-diastolic frames were averaged for each resting baseline measurement (R) and from at least three frames at maximum dilation during reactive hyperemia (H). Change in brachial artery diameter (ΔBAD) and FMD %, our primary outcome, were calculated as:

$$\Delta \text{BAD}=\text{H}-\text{R};\text{FMD}\%=\left(\left(\text{H}-\text{R}\right)/\text{R}\right)\times 100.$$

Blood pressure (BP) was measured by trained nursing staff shortly after arrival to the study center. Measurements were performed according to recommended timing and positioning and using the appropriate BP cuff size by a validated automated office-type oscillometric device [24]. Three measurements were taken and averaged to determine the participant's office BP measurement for the visit. Ambulatory blood pressure measurements used for enrollment into the study were recorded using an Oscar 2 ambulatory BP monitor programmed to record blood pressure at 30-minute intervals from 6 am to 11 pm and at hourly intervals during the remainder of the day.

### Side Effects, Adherence, and Acceptability

Side effects and adverse events were evaluated at each weekly follow-up visit using a symptom checklist that was administered by the study coordinator. Symptom severity was self-graded as mild, moderate, or severe, and further details were elicited in free form.

Adherence to the dosing regimen was measured using participant self-report. Participants were asked to return any unused doses at their next study visit.

To evaluate how participants thought they might use hawthorn if it were proven to be effective for reducing blood pressure, we asked four questions: (1) how likely would you be to take hawthorn instead of lifestyle modification?; (2) how likely would you be to take hawthorn in conjunction with lifestyle modification?; (3) how likely would you be to take hawthorn instead of a prescription antihypertensive medication?; and (4) how likely would you be to take hawthorn in conjunction with a prescription antihypertensive medication? Answers were recorded on a 5-point likert scale from 'very likely' to 'very unlikely' with 'unsure' at the center of the scale.

### Analysis

Our primary outcome was FMD %. We tested the dose-response effect using a modified linear contrast [25] in a mixed effects linear regression model that included random subject effects. The model included fixed effects for dose, period, resting BAD within each period, and baseline FMD %. We first assessed the potential for carry-over effects by including dummy indicators for dose used in the prior period (all indicators were zero for the first period and for periods following placebo) and conducting a 3 degree of freedom test at the 0.05 level. If non-significant, the carry-over indicators were removed, and the modified linear contrast was tested at the 0.05 level. If a significant dose-response effect was observed, the pre-specified plan was pairwise testing of each dose, in descending order, versus placebo until a non-significant comparison was observed. Similar methods were used to assess dose-response effects on ΔBAD, resting BAD, systolic and diastolic BP, and hyperemic VTI. Given published information on the variance and covariance of repeated FMD measurements and assuming that a 2500 mg dose would lead to a 1% increase in mean FMD levels, we calculated that obtaining complete data on at least 20 participants would provide at least 80% power to detect a dose-response relationship even if there were no effects at the lower doses [26]. All analyses were conducted in SAS, Version 9.2 (SAS Institute, Cary, NC).

## Results

**Figure 1:**
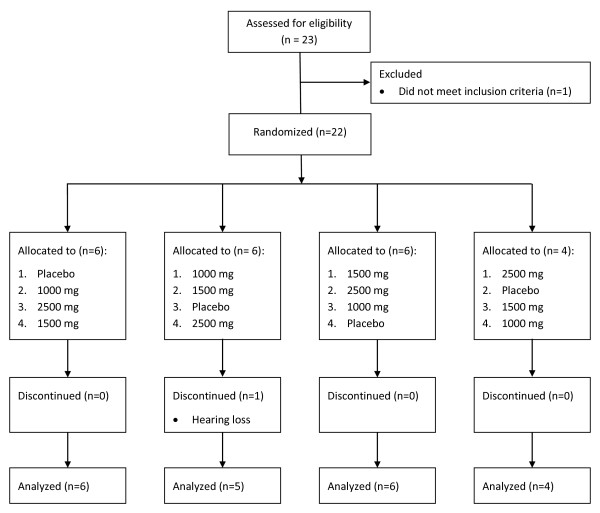
**Study participant flow chart**. Four random dose sequences were created, and each individual dose was considered a dosing period. Participants were randomly assigned to a single sequence.

We enrolled 22 participants with baseline systolic/diastolic ambulatory blood pressure (ABP) between 120-155/80-95 mm Hg from the University of North Carolina at Chapel Hill catchment area between July 2010 and May 2011 (Figure 1). Twenty-one participants fully completed the study and were included in the analyses. Participation was halted for one subject due to hearing loss. The mean age of participants was 51 years (range 35 - 78 years), and the mean (SD) systolic ABP was 137.6 (7.7) mm Hg and mean (SD) diastolic ABP was 81.8 (6.3) mm Hg (Table 1).

**Table 1:** Baseline Characteristics (N = 21)

	**Mean (SD) or n (%)**
**Age**, years	50.9 (10.5)

**BMI**	29.3 (5.4)

**Ambulatory BP**, mm Hg	
Systolic	137.6 (7.7)
Diastolic	81.8 (6.3)

**Baseline FMD**	
FMD %	4.3 (2.8)
Δ BAD, mm	0.15 (0.09)

**Race**	
White	15 (71.4%)
Black	6 (28.6%)

**Gender**	
Male	9 (42.8%)
Female	12 (57.2%)

**Marital Status**	
Single	10 (47.7%)
Married	11 (52.3%)

**Self-reported Health Status**	
Excellent, Very Good, Good	21 (100%)
Fair or Poor	0

BMI - body mass index, BP - blood pressure, FMD - flow mediated dilation, BAD - brachial artery diameter

### Flow-mediated dilation, brachial artery diameter, velocity-time integral, and blood pressure

No evidence of carry-over effects for any outcome (p ≥ 0.23 for all models) was observed. There was no evidence of a dose-response effect for our primary outcome, FMD % (p = 0.59) (Table 2). Mean change in brachial artery diameter (BAD) ranged from 0.14 mm - 0.18 mm, with no evidence of a dose-response effect (p = 0.54). The mean (SD) peak systolic velocity (cm/s) during hyperemia was 155.3 (22.8), with minimum and maximum velocities of 106 and 224, respectively. There was also no evidence of a dose-response effect for resting BAD (p = 0.74) or mean resting BAD by study period (p = 0.50, data not shown). The mean (SD) hyperemic VTI was 85.7 cm (19.9) with a range of 46.0 - 135.0 cm. Among the four groups, the mean (SD) ranges of systolic and diastolic BP were 129.4 (8.1) - 132.0 (10.0) mm Hg and 81.8 (7.8) - 83.0 (9.1) mm Hg, respectively, with no evidence of dose-response effects (p = 0.33 and 0.72, respectively).

**Table 2:** Mean flow-mediated dilation, brachial artery diameter, velocity-time integral, and blood pressure by dose (N = 21)

	**Placebo**	**1000 mg**	**1500 mg**	**2500 mg**	***p*-value^1^**
**FMD**					
Δ BAD, mm	0.18 (0.14)	0.16 (0.12)	0.14 (0.13)	0.17 (0.12)	0.54
FMD, %	5.3 (4.3)	5.0 (4.1)	4.3 (4.0)	4.9 (4.1)	0.59

**BAD**, mm					
Resting	3.5 (0.7)	3.5 (0.8)	3.6 (0.8)	3.5 (0.8)	0.74
Hyperemia	3.7 (0.7)	3.6 (0.8)	3.7 (0.8)	3.7 (0.8)	-

**VTI**, cm	85.7 (20.8)	91.3 (19.5)	82.0 (21.4)	83.9 (18.0)	0.31

**Blood Pressure**, mm Hg					
Systolic	129.4 (8.1)	130.7 (10.7)	131.1 (13.6)	132.0 (10.0)	0.33
Diastolic	82.8 (7.3)	81.8 (7.8)	83.0 (9.1)	82.3 (7.7)	0.72

^1 ^*p*-value for modified linear contrast assessing dose-response from mixed effects linear model controlling for period and baseline response. Models for FMD also controlled for resting BAD.

All values expressed as mean (SD).

FMD - flow mediated dilation, BAD - brachial artery diameter, VTI - velocity-time integral

To explore the possibility that participants with higher and lower baseline blood pressure may respond differently to HSE, we added to the primary model interactions between doses of HSE and an indicator for baseline systolic ABP either above or below the median (129 mm Hg). We found no evidence of an interaction (p = 0.16).

### Side effects

Side effects were uncommon and the rates of events for placebo versus HSE were typically similar. The most common side effects were mild nausea (9.5% vs. 7.9%, placebo vs. HSE), mild to moderate headache (14.3% vs. 15.9%), and mild palpitations (4.7% vs. 7.9%). Dizziness occurred in only a single participant taking placebo. One participant reported a fall (2500 mg dose) while climbing an icy staircase, and one participant reported partial hearing loss (during the 1000 mg dose period) but had prior instances of idiopathic hearing loss.

### Adherence, blinding, and acceptability

Overall, 78 of the 84 dose sheets (93%) were returned, all of which indicated that doses had been taken twice a day as directed. When the dose sheet was forgotten, we asked participants if they had taken their pills as directed, and all participants responded affirmatively. One participant reported taking two doses on the same morning instead of split AM and PM. One unused dose cup was returned by a single participant.

To assess blinding, we asked participants to guess in which dosing period they had received placebo pills. Eleven participants guessed incorrectly, one guessed correctly, and nine did not guess (we would have expected 25% to guess correctly by chance).

Most participants answered that they were likely or very likely to use hawthorn with or instead of either lifestyle modification (91% and 67%, respectively) or prescription medication (57% and 86%, respectively) if given proof that hawthorn could lower blood pressure (Table 3).

**Table 3:** Likelihood to use Hawthorn for blood pressure control

***How likely would you be to use Hawthorn for blood pressure lowering: ***(N = 21)		
	**instead of:** **n (%)**	**in conjunction with:** **n (%)**

**lifestyle modification^1^**		
Likely	14 (66.6)	19 (90.5)
Unsure	4 (19.0)	0
Unlikely	3 (14.4)	2 (9.5)

**antihypertensive medication**		
Likely	18 (85.7)	12 (57.2)
Unsure	2 (9.5)	5 (23.8)
Unlikely	1 (4.8)	4 (19.0)

^1^Lifestyle modification defined as dietary and/or exercise modifications.

## Discussion

Hawthorn and some of its individual constituents (e.g., oligomeric procyanidins [OPC], flavonoids) have been shown to have effects on nitric oxide (NO) and endothelial function, which are implicated in hypertension and CVD [27,28]. Hawthorn has been shown to produce endothelial-dependent arterial vasodilation in mice, rats, pigs, and humans [3,4,29,30] that is linked to activation of endothelial NO-synthase (NOS) [3]. Studies of different fractions of HSE have demonstrated that it is the OPC-rich fraction that mediates hawthorn's vasodilatory effects [3,4]. Therefore, we sought to investigate the effects of a standardized, OPC-concentrated hawthorn formulation on flow-mediated brachial artery dilation, a measure of NO release. However, we did not detect a dose-response effect on FMD, and no dose appeared to have an effect significantly different than placebo, indicating that HSE may not have a clinically meaningful effect on NO in humans.

Formation of endogenous NO is typically mediated by NOS through the oxidation of the amino acid L-arginine. It is now understood that profound reduction of NO occurs with aging so that a 40 year old adult may only produce half as much NO as a 20 year old [31-33]. However, strategies to increase NO production by supplementing with L-arginine have failed to demonstrate any clinically meaningful results in human trials [34], leading to the conclusion that another pathway may need to be activated to increase NO production.

Exogenous intake of nitrate in humans is converted by commensal bacteria to nitrite, which can then be reduced to NO [35]. However, the reduction process catalyzed by nitrite reductase is particularly inefficient [36]. Zand, et al. screened over 100 botanicals and found hawthorn to have the highest nitrate reductase activity. They then tested a combination supplement that was both rich in natural nitrate (beetroot) and nitrite reductase activity (hawthorn) and found that the supplement produced sustained *in vitro *NO release with a t_1/2 _of about 60 minutes and rapid and sustained human plasma NO levels (~1.4 μM) after a single dose *in vivo*. Steady state human plasma nitrite concentrations rose from 0.10 μM to 0.28 μM after taking the supplement for 30 days, which was statistically significantly greater than placebo, while plasma nitrite concentrations decreased in the placebo group. Finally, mildly hypertensive participants (systolic BP 135 - 160 mm Hg, n = 9) experienced a mean 7 mm Hg systolic and 2.7 mm Hg diastolic blood pressure reduction, although this was not statistically significant [35].

Given what is known about the pathways of NO production, it is possible that hawthorn alone is insufficient to have a meaningful effect on NO and may require addition of nitrate/nitrite to be effective *in vivo*. We did not measure plasma nitrate concentration in the current study; additionally, the mean age of our participants was 51 years and nearly all participants were older than 40 years, so the ability to produce NO was likely limited in our study population. Therefore, our study results may reflect a poor native ability to produce NO.

Studies of hawthorn to lower BP in humans suggest benefits, although only three small randomized, placebo-controlled trials have been performed [9-11]. Asgary, et al. treated mildly hypertensive adults for 16 weeks and observed mean systolic and diastolic BP reductions of 13 mm Hg and 8 mm Hg respectively. Walker, et al. studied both untreated mildly hypertensive patients and treated hypertensive diabetic patients for 10-16 weeks and observed systolic BP reductions of 3.6-10 mm Hg and diastolic BP reductions of 2.6-7 mm Hg. Other studies of the effect of hawthorn on heart failure also suggest that it may have a BP lowering effect, although BP was a secondary outcome in each of those studies [19,20]. All hawthorn studies to lower BP have had a minimum 10 week intervention period and the German Commission E recommends a minimum of 6 weeks [37]. Even if hawthorn has no effect on NO, it is still worthwhile to investigate the effects of hawthorn on blood pressure given the results of these prior trials.

### Limitations

Several limitations of this study should be considered. Although we measured adherence by self-report, poor adherence could have masked any effect of hawthorn, and we did not include a pharmacologic adherence assessment. However, no individual participants exhibited a dose-response effect, and it would be very unlikely that all participants were uniformly poorly adherent. Pharmacokinetic parameters of hawthorn constituents are poorly described for humans. Reports in rodents demonstrate poor oral absorption and bioavailability for some constituents of hawthorn [21]. Poor absorption of the HSE pills could confound our results, but would not contradict preclinical *in vitro *and *ex vivo *findings. However, similar doses of HSE have been used for trials in patients with HF that have shown beneficial effects, suggesting there is sufficient oral bioavailability of HSE to demonstrate physiologic effects. It is possible that twice daily dosing of HSE for 4 days is insufficient to have an effect on NO production. However, most mechanisms to increase NO production in response to shear stress, such as that produced during FMD, take minutes to hours to occur (eg. calcium ion flux, eNOS activation, eNOS gene transcription) [23].

Little is known about differences among HSE formulations so our results may not be generalizable to other hawthorn formulations. The extract for this trial was a whole leaf and flower formulation with sufficient plant material to provide OPC at high concentrations. Other extraction techniques may favor a different mix of constituents (ie. hydrophilic vs hydrophobic extraction processes), as would doping a low OPC extract with additional OPC-rich fraction to obtain high OPC concentrations.

## Conclusion

We found no evidence that flow mediated dilation, an indirect measure of NO, is altered by multidose administration of hawthorn standardized extract. If there is a blood pressure lowering effect of hawthorn, it is likely to occur by mechanisms other than NO-mediated vasodilation.

## Competing interests

Hawthorn standardized extract and placebo capsules were generously donated by Gaia, Inc.

## Authors' contributions

GNA conceived of the study, participated in its design and coordination, and drafted the manuscript. AJV participated in the design of the study and helped to draft the manuscript. MAW performed the statistical analysis and edited the manuscript. RD participated in the design of the study. MC gathered the FMD data. ALH participated in the study design and coordination, and helped draft the manuscript. All authors read and approved the final manuscript.

## Pre-publication history

The pre-publication history for this paper can be accessed here:

http://www.biomedcentral.com/1472-6882/12/26/prepub

## References

[B1] Upton R Hawthorn Berry American Herbal Pharmacopoeia 1999 Scotts Valley: AHP 24

[B2] Chang Q Zuo Z Harrison F Chow MS Hawthorn J Clin Pharmacol 2002 42 6 605 612 10.1177/00970002042006003 12043949

[B3] Brixius K Willms S Napp A Tossios P Ladage D Bloch W Mehlhorn U Schwinger RH Crataegus special extract WS 1442 induces an endothelium-dependent, NO-mediated vasorelaxation via eNOS-phosphorylation at serine 1177 Cardiovasc Drugs Ther 2006 20 3 177 184 10.1007/s10557-006-8723-7 16779533

[B4] Kim SH Kang KW Kim KW Kim ND Procyanidins in crataegus extract evoke endothelium-dependent vasorelaxation in rat aorta Life Sci 2000 67 2 121 131 10.1016/S0024-3205(00)00608-1 10901280

[B5] Koch E Chatterjee S Crataegus extract WS 1442 enhances coronary flow in the isolated rat heart by endothelial release of nitric oxide Naunyn Schmiedebergs Arch Pharmacol 2000 361 Suppl R48

[B6] Ellingwood F JU L: American Materia Medica, Therapeutics and Pharmacognosy 1915 Evanston: Ellingwood's Therapeutist

[B7] ESCOP Monographs 2003 2 Stuttgart: Thieme

[B8] Guo R Pittler MH Ernst E Hawthorn extract for treating chronic heart failure Cochrane Database Syst Rev 2008 1 CD005312 18254076 10.1002/14651858.CD005312.pub2 PMC11753770

[B9] Asgary S Naderi GH Sadeghi M Kelishadi R Amiri M Antihypertensive effect of Iranian Crataegus curvisepala Lind.: a randomized, double-blind study Drugs Exp Clin Res 2004 30 5-6 221 225 15700749

[B10] Walker AF Marakis G Morris AP Robinson PA Promising hypotensive effect of hawthorn extract: a randomized double-blind pilot study of mild, essential hypertension Phytother Res 2002 16 1 48 54 10.1002/ptr.947 11807965

[B11] Walker AF Marakis G Simpson E Hope JL Robinson PA Hassanein M Simpson HC Hypotensive effects of hawthorn for patients with diabetes taking prescription drugs: a randomised controlled trial Br J Gen Pract 2006 56 527 437 443 16762125 PMC1839018

[B12] Chen JD Wu YZ Tao ZL Chen ZM Liu XP Hawthorn (shan zha) drink and its lowering effect on blood lipid levels in humans and rats World Rev Nutr Diet 1995 77 147 154 7732698 10.1159/000424470

[B13] Rajendran S Deepalakshmi PD Parasakthy K Devaraj H Devaraj SN Effect of tincture of Crataegus on the LDL-receptor activity of hepatic plasma membrane of rats fed an atherogenic diet Atherosclerosis 1996 123 1-2 235 241 10.1016/0021-9150(96)05813-3 8782854

[B14] Shanthi S Parasakthy K Deepalakshmi PD Devaraj SN Hypolipidemic activity of tincture of Crataegus in rats Indian J Biochem Biophys 1994 31 2 143 146 7927437

[B15] Shanthi S Parasakthy K Deepalakshmi PD Niranjjali D Protective effect of tincture of Crataegus on wxidative stress in experimental atherosclerosis in rats J Clin Biochem Nutr 1996 20 211 223 10.3164/jcbn.20.211

[B16] Zhang Z Ho WK Huang Y James AE Lam LW Chen ZY Hawthorn fruit is hypolipidemic in rabbits fed a high cholesterol diet J Nutr 2002 132 1 5 10 11773500 10.1093/jn/132.1.5

[B17] Moens AL Goovaerts I Claeys MJ Vrints CJ Flow-mediated vasodilation: a diagnostic instrument, or an experimental tool? Chest 2005 127 6 2254 2263 10.1378/chest.127.6.2254 15947345

[B18] Tauchert M Efficacy and safety of crataegus extract WS 1442 in comparison with placebo in patients with chronic stable New York Heart Association class-III heart failure Am Heart J 2002 143 5 910 915 10.1067/mhj.2002.121463 12040357

[B19] Tauchert M Gildor A Lipinski J [High-dose Crataegus extract WS 1442 in the treatment of NYHA stage II heart failure] Herz 1999 24 6 465 474 discussion 475 10.1007/BF03044432 10546150

[B20] Weikl A Assmus KD Neukum-Schmidt A Schmitz J Zapfe G Noh HS Siegrist J [Crataegus Special Extract WS 1442. Assessment of objective effectiveness in patients with heart failure (NYHA II)] Fortschr Med 1996 114 24 291 296 8974970

[B21] Chang Q Zuo Z Ho WK Chow MS Comparison of the pharmacokinetics of hawthorn phenolics in extract versus individual pure compound J Clin Pharmacol 2005 45 1 106 112 10.1177/0091270004270500 15601812

[B22] Wang B Wang X Gong L The Construction of a Williams Design and Randomization in Cross-Over Clinical Trials Using SAS Journal of Statistical Software 2009 29 Code snippet 1, pp1-10

[B23] Corretti MC Anderson TJ Benjamin EJ Celermajer D Charbonneau F Creager MA Deanfield J Drexler H Gerhard-Herman M Herrington D Guidelines for the ultrasound assessment of endothelial-dependent flow-mediated vasodilation of the brachial artery: a report of the International Brachial Artery Reactivity Task Force J Am Coll Cardiol 2002 39 2 257 265 10.1016/S0735-1097(01)01746-6 11788217

[B24] Pickering TG Hall JE Appel LJ Falkner BE Graves J Hill MN Jones DW Kurtz T Sheps SG Roccella EJ Recommendations for blood pressure measurement in humans and experimental animals: Part 1: blood pressure measurement in humans: a statement for professionals from the Subcommittee of Professional and Public Education of the American Heart Association Council on High Blood Pressure Research Hypertension 2005 45 1 142 161 15611362 10.1161/01.HYP.0000150859.47929.8e

[B25] Dmitrienko A Fritsch K Hsu J Ruberg S Dmitrienko A, Chuangstein C, Ralph D'Agostino S Design and analysis of dose-ranging clinical studies Pharmaceutical Statistics Using SAS: A Practical Guide 2007 Cary: SAS Institute, Inc 274 311

[B26] Donald AE Halcox JP Charakida M Storry C Wallace SM Cole TJ Friberg P Deanfield JE Methodological approaches to optimize reproducibility and power in clinical studies of flow-mediated dilation J Am Coll Cardiol 2008 51 20 1959 1964 10.1016/j.jacc.2008.02.044 18482664

[B27] Panza JA Quyyumi AA Brush JE Jr Epstein SE Abnormal endothelium-dependent vascular relaxation in patients with essential hypertension N Engl J Med 1990 323 1 22 27 10.1056/NEJM199007053230105 2355955

[B28] Shimbo D Grahame-Clarke C Miyake Y Rodriguez C Sciacca R Di Tullio M Boden-Albala B Sacco R Homma S The association between endothelial dysfunction and cardiovascular outcomes in a population-based multi-ethnic cohort Atherosclerosis 2007 192 1 197 203 10.1016/j.atherosclerosis.2006.05.005 16762358

[B29] Chen ZY Zhang ZS Kwan KY Zhu M Ho WK Huang Y Endothelium-dependent relaxation induced by hawthorn extract in rat mesenteric artery Life Sci 1998 63 22 1983 1991 10.1016/S0024-3205(98)00476-7 9839542

[B30] Vierling W Brand N Gaedcke F Sensch KH Schneider E Scholz M Investigation of the pharmaceutical and pharmacological equivalence of different Hawthorn extracts Phytomedicine 2003 10 1 8 16 10.1078/094471103321648601 12622458

[B31] Egashira K Inou T Hirooka Y Kai H Sugimachi M Suzuki S Kuga T Urabe Y Takeshita A Effects of age on endothelium-dependent vasodilation of resistance coronary artery by acetylcholine in humans Circulation 1993 88 1 77 81 8319359 10.1161/01.cir.88.1.77

[B32] Gerhard M Roddy MA Creager SJ Creager MA Aging progressively impairs endothelium-dependent vasodilation in forearm resistance vessels of humans Hypertension 1996 27 4 849 853 8613259 10.1161/01.hyp.27.4.849

[B33] Taddei S Virdis A Ghiadoni L Salvetti G Bernini G Magagna A Salvetti A Age-related reduction of NO availability and oxidative stress in humans Hypertension 2001 38 2 274 279 11509489 10.1161/01.hyp.38.2.274

[B34] Schulman SP Becker LC Kass DA Champion HC Terrin ML Forman S Ernst KV Kelemen MD Townsend SN Capriotti A L-arginine therapy in acute myocardial infarction: the Vascular Interaction With Age in Myocardial Infarction (VINTAGE MI) randomized clinical trial JAMA 2006 295 1 58 64 10.1001/jama.295.1.58 16391217

[B35] Zand J Lanza F Garg HK Bryan NS All-natural nitrite and nitrate containing dietary supplement promotes nitric oxide production and reduces triglycerides in humans Nutr Res 2011 31 4 262 269 10.1016/j.nutres.2011.03.008 21530799

[B36] Feelisch M Fernandez BO Bryan NS Garcia-Saura MF Bauer S Whitlock DR Ford PC Janero DR Rodriguez J Ashrafian H Tissue processing of nitrite in hypoxia: an intricate interplay of nitric oxide-generating and -scavenging systems J Biol Chem 2008 283 49 33927 33934 10.1074/jbc.M806654200 18835812 PMC2590701

[B37] Blumenthal M Goldberg A Brinckmann J Herbal Medicine: Expanded Commission E Monographs 2000 Newton, MA: IntegrativMedicine

